# Non-Destructive Ellipsometric Analysis of the Refractive Index of Historical Enamels

**DOI:** 10.3390/ma18051137

**Published:** 2025-03-03

**Authors:** Teresa Palomar, Trinitat Pradell, Jadra Mosa

**Affiliations:** 1 VICARTE Research Unit, NOVA School of Science & Technology, Campus Caparica, 2829-516 Caparica, Portugal; 2 Institute of Ceramic and Glass (ICV-CSIC), c/ Kelsen 5, Campus de Cantoblanco, 28049 Madrid, Spain; jmosa@icv.csic.es; 3 Departament de Física, Centre de Recerca en Ciència i Enginyeria Multiescala de Barcelona, Universitat Politècnica de Catalunya·BarcelonaTech (UPC), Campus Diagonal Besòs, Av. Eduard Maristany, 10-14, 08019 Barcelona, Spain; trinitat.pradell@upc.edu

**Keywords:** refractive index, ellipsometry, historical enamels, glass

## Abstract

The refractive index is an important parameter for the restoration of historical cultural heritage and for non-destructive optical techniques. In this study, different mathematical models for lead glasses were assessed in order to analyze their feasibility to calculate the theoretical refractive index of the historical enamels in stained-glass windows. The models selected were those specifically developed for lead glasses: the Appen method (1949), the Fanderlik and Skrivan model (1972), and the Bonetti and Salvagno method (1983). The results of the mathematical methods were compared with the real values analyzed via ellipsometry. The historical enamels were determined on non-prepared samples, taking into account the Cauchy model in order to avoid damaging the historical pieces. We show that the measured refractive indices of the historical enamels (1.59–1.66) are higher than the values of the lead glasses in the literature (1.55–1.57). The PbO and B_2_O_3_ were the compounds that most influenced the value of the refractive index; nevertheless, the presence of metallic elements increased their value compared to pure lead glasses. In addition, the presence of a thin layer of grisaille on the historical enamels and the formation of alteration layers could also modify the real value of the refractive index. As far as theoretical calculations are concerned, the mathematical model of Bonetti and Salvagno (1983) seems to be the most accurate model for this material, with errors < 0.04 units. None of the three models work for glasses with >60% PbO, which is not common in cultural heritage.

## 1. Introduction

The refractive index (n) is one of the optical properties of glasses, along with transparency and coloration. It is manifested when a ray of light enters in a glass from the air, where it possesses the maximum velocity, and its velocity is reduced as a result of the interaction of the light with the ions in the glass. In the case of the vertical incidence of light, the path of the light ray is not altered, but in the case of oblique incidence, a deviation occurs [1].

The refractive index is related to the electronic density in the glass and the polarizability of its constituent elements. The bonding oxygens in the glass matrix are strongly polarized and poorly deformable, so the refractive index is low. The addition of modifier oxides, such as alkaline and alkaline-earth elements, increases the number of non-bonding oxygens and, hence, the polarizability of the oxygen and the refractive index of the glass. The polarization of cations, especially those with small ionic radii, is very low compared to anions. Voluminous cations with partially filled outer shells significantly increase the refractive index [2].

The refractive index has also been used for new materials with optical properties [3], solar concentrators [4,5], smart windows [6,7], or to imitate gemstones [8]. In forensic investigations, the chemical composition and refractive index of glass micro-fragments are also analyzed to determine their origin (i.e. automobile windows, headlamps, side mirrors, beverage container glasses…) [9].

Few works have analyzed the refractive index of historical glasses, probably because it is necessary to prepare a sample with a moderate size, which should not be allowed in cultural heritage objects. Tennent and Townsend [10] compiled 53 analyses of historical glasses from different museums and collections. They were clustered in four groups. The Roman and Egyptian glasses showed 1.490 < n < 1.540, the post-Medieval vessel glasses had 1.505 < n < 1.530, the Medieval stained glasses presented 1.540 < n < 1.585, and the lead glasses were 1.550 < n < 1.570. Vassas [11] also analyzed Medieval stained glasses, whose values ranged between 1.5102 < n < 1.5331 for soda-lime silicate glasses, 1.5379 < n < 1.5514 for potash-lime silicate glasses, and 1.5546 < n < 1.5670 for high-lime low-alkali glasses, agreeing with the Tennent and Townsend groups. Saminpanya et al. [12] analyzed ancient Indo-Pacific beads made of soda-alumina glasses, whose values ranged from 1.51 to 1.60, with this variation related to their coloration and, therefore, to their chemical composition. Further, Drozdov et al. [13] analyzed some opaque potassium lead silicate glasses for mosaics and bijouterie produced by Mikhail Lomonosov in the Ust-Ruditsa factory in the 18th century. Their refractive indices ranged from 1.493 to 1.825. Glasses with high lead content have higher refractive indices. The refractive index is an important parameter in glass restorations [10,14,15]. The adhesive should have a similar refractive index to conceal the fissures’ reparation. The closer the refractive index of the adhesive is to that of the glass, the less noticeable the repair will be.

It is also important in optical techniques used for the non-destructive analysis of cultural heritage. Optical coherence tomography (OCT) uses the reflection and scattering of light to identify the multilayer structure and to measure the thickness of the different layers. The tomograms are corrected for the refractive index of the materials penetrated by the probing beam, assuming that the refractive index remains relatively constant in space and time [16,17]. Another example is nonlinear optical microscopy (NLOM), which provides compositional and structural information based on the detection of the emission fluorescence of fluorophores through multiphoton excitation fluorescence microscopy (MPEF) and local differences in refractive indices via third-harmonic generation microscopy (THG) [18,19]. Three-dimensional Confocal Microscopy also allows measurements to be made, considering that the refractive index mismatch between the immersion fluid and sample can affect the focus position and, therefore, making axial distances appear more elongated [20]. Terahertz time-domain spectroscopy (THz-TDS) uses THz pulses to epi-detect the signal reflected from the crossed interfaces between materials characterized by different refractive indices, allowing cross-sectional images of the object’s stratigraphy to be reconstructed [18].

These techniques have been widely applied to cultural heritage objects. OCT has been applied to characterize the surface alteration in glass objects [21,22,23,24,25]; NOLM has been used to determine the thickness of alteration layers in medieval-like glasses [26] and historical glass grisailles [27,28]; and Confocal Microscopy has allowed for the measurement of the thickness of laminated altered layers in historical glasses [29]. The refractive index of glasses and their decoration is, therefore, a key parameter for their characterization and restoration. Several techniques have been developed to measure the refractive index. The most common methods are the deflection of a beam of light in a prism with an angle of about 60°; the refractometer, in which a glass polished on one side is pressed onto the refractometer prism with a liquid of a high index of refraction; the immersion method, in which small glass pieces are immersed in liquids of known refractive indices until the glass “disappears”; or with an interferometer that detects the interference produced by the glass on one of the two light beams emitted by the instrument [1,2]. Most of these techniques require sample preparation and sufficient size (usually a few centimeters) and are, therefore, not suitable for historical glasses. Another method of measuring the refractive index is ellipsometry, which is based on the measurement of the elliptical polarization experienced by a beam of polarized light when reflected from the surface of a glass [1,2]. This technique permits the non-destructive analysis of surface layers, even those only a few nanometers thick. For this reason, this technique should be suitable for cultural heritage material. In this study, ellipsometry was employed to determine the refractive indices of historical enamel samples. Ellipsometric measurements are based on the changes in the polarization of light reflected from the sample surface, from which information about the optical properties of the sample is extracted. However, obtaining reliable refractive index values requires careful analysis of the ellipsometric data through advanced modeling techniques. Specialized software tools were used to fit the experimental ellipsometric data to models describing the optical behavior of the enamel films. These models take into account various parameters, such as layer thickness, roughness, and the refractive index itself, among others. In this regard, the refractive index of surface enamels from stained-glass windows is very complicated to measure because they are highly fusible colored glasses that are applied as surface paintings [30], and lead and/or borax (Na_2_B_4_O_7_·10H_2_O) are usually used to reduce the melting temperature [31]. Therefore, ellipsometry is proposed as a good technique to carry out these measurements.

The main objective of this study is to determine the refractive index of the enamel layers (high-lead glasses) on historical glasses. For this purpose, different mathematical models from the bibliography for silicate glasses [32,33] and lead glasses [34,35] were evaluated for glasses with chemical compositions similar to historical enamels. The feasibility of applying non-destructive ellipsometry to historical enamels was also assessed, and, finally, the agreement between mathematical and the experimental results was evaluated.

## 2. Materials and Methods

### 2.1. Samples

**Figure 1:**
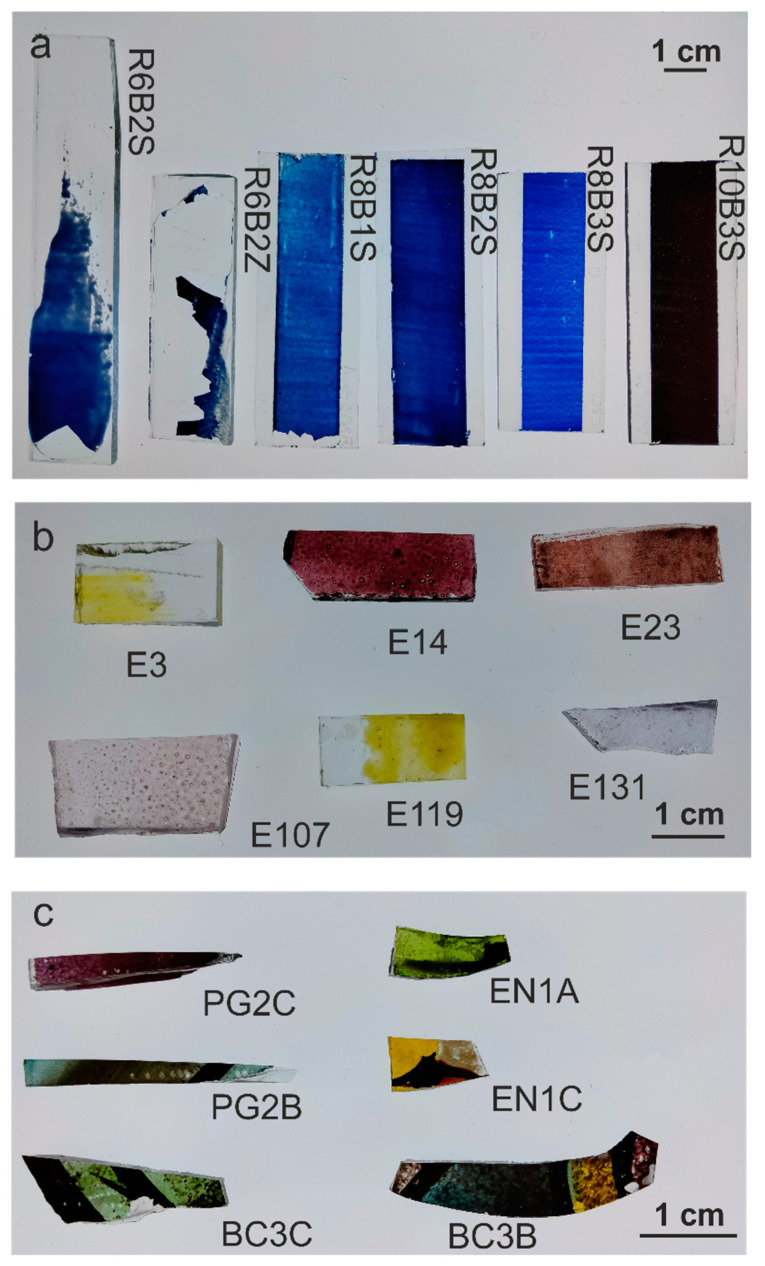
Sample sets: (**a**) Set 1: produced enamels; (**b**) set 2: historical enamels painted; (**c**) set 3: historical enamels from stained-glass windows.

Three sets of samples were analyzed. The first set (Figure 1a), produced in VICARTE laboratories, consisted of blue enamels prepared according to the recipes of the historical treatise The Handmaid to the Arts (1758) by Robert Dossie [31]. The second set (Figure 1b), produced by J.M. Bonet Vitralls S.L., was prepared using historical enamels from the Rigalt, Granell & cia workshop [36] on modern glass. And the third set (Figure 1c) consisted of historical pieces of Catalan Modernist stained-glass windows from the beginning of the 20th century [37]. The samples were analyzed without any preparation in order to preserve the historical material. To minimize measurement errors, each enamel was measured 3 times in each area, and each sample was analyzed in at least 3 areas (n > 9).

### 2.2. Theoretical Calculations

Enamels are glasses that melt at a lower temperature than the glass to which they are applied [30]. Lead and/or borax (Na_2_B_4_O_7_·10H_2_O) are usually used to lower the melting temperature [31]. As the refractive index is directly influenced by the ions in the glass matrix, 33 lead borosilicate glasses (Bansal and Doremus, 1986) [38], 34 lead silicate glasses (Bonetti and Salvagno, 1983) [35] and 16 lead silicate glasses (Fanderlik and Skrivan, 1972) [34] were considered for the assessment of the feasibility of the proposed methods.

Three mathematical methods were assessed. The Appen method was proposed for silicate glasses. In it, the refractive index is calculated from the portions of the individual elements by using Equation (1) and the factors contained in (Appen, 1949) [32,33]. *p_i_* is the molar percentage (mol %) of each oxide and n_i_ is their specific factor.

(1) $$n=\frac{\sum n_{i}\cdot p_{i}}{100}$$

To simplify the calculations, Fanderlik and Skrivan [34] formulated an equation to calculate the refractive index of the “sonoro superiore” lead glasses, in which the percentage of PbO is between 0 and 5 wt.% (Equation (2)). Some years later, Bonetti and Salvagno [35] developed another equation for glasses with a PbO content between 21 and 27 wt.% (Equation (3)). In these two models, the p_i_ is the weight percentage (wt.%).

(2) $$n_{D}=1.46037+0.00079p_{{Na}_{2}O}+0.00290p_{CaO}+0.00167p_{BaO}+0.00228p_{MgO}+0.00327p_{ZnO}+0.00076p_{{Al}_{2}O_{3}}\;+0.00141p_{B_{2}O_{3}}+0.00337p_{PbO}+0.000053p_{PbO}\cdot p_{CaO}+0.000068p_{PbO}\cdot p_{BaO}$$

(3) $$n_{D}=1.46221+0.00151p_{{Na}_{2}O}+0.0011p_{K_{2}O}+0.00316p_{CaO}+0.0023p_{BaO}+0.00198p_{ZnO}+0.00258p_{PbO}\;+0.00215p_{B_{2}O_{3}}\;$$

### 2.3. Analytical Methods

Enamels were measured via ellipsometry to determine their refractive index. Spectral ellipsometric measurements were performed using a Variable Angle Spectroscopic Ellipsometer (WVASE32, M-2000UTM, J.A. Co., Woollam, Lincoln, NE, USA) to characterize the refractive index of glazes deposited onto glass slides. Spectra were recorded in the visible range between 250 and 900 nm at variable angles of incidence of 65°, 70° and 75° and photon energies in the range of 0.7–4.0 eV (1770–310 nm wavelength). The illuminated area of the sample at these angles is approximately 3 × 7 mm^2^. The data were fitted using the WVASE32 software and considering the Cauchy model [39]. WVASE32 employs sophisticated fitting algorithms to minimize the difference between the measured and modelled data, allowing for accurate extraction of optical constants. The software can handle measurements taken at multiple angles of incidence, which is critical for accurate modelling of optical properties. Different optical models are available, including single-layer, multi-layer, and complex models, depending on the sample structure. Among these, the Cauchy model describes the refractive index of a transparent material as a function of wavelength (λ) and is particularly suitable for materials that are transparent in the visible to near-infrared range, making it ideal for glass and enamel. The model is relatively simple and requires fewer parameters than more complex models, making it easier to fit and interpret.

The data collected from the samples were first fitted with a transparent Cauchy layer to provide an initial value of n and e. Using this thickness, the data were then fitted by varying the refractive index and extinction coefficient (k) of the layer; in all cases, only very small values of k (weak absorption) were obtained. These best-fit values were then parameterized with a general oscillator model to ensure Kramers–Kronig consistency of the optical model [40]. Specifically, the model can include multiple layers to account for glass and the enamel coating. Each layer presents its own refractive index and thickness. Calibration was performed using standard silicon as reference material with known optical properties to ensure accurate measurements and then aligning the optical components and verifying the zero point before each measurement. Measurements were performed at a constant humidity level (typically around 40%) to prevent moisture from affecting the measurements and at a temperature (around 25 °C) to minimize thermal effects on the measurements. Samples were thoroughly cleaned with lint-free wipes to remove any contaminants and then handled carefully to avoid scratches or other damage. WVASE32 was used for data acquisition and analysis, allowing the ellipsometry data to be fitted to a selected appropriate optical model. Experimental data were fitted to an optical model, and thickness and refractive index were adjusted to achieve the best fit. Historical enamels can exhibit significant heterogeneity in their chemical composition due to variations in raw materials and manufacturing processes. This can affect their optical properties and may result in the sample being analyzed in different zones. The mean and standard deviation of the measured ellipsometry angles (Ψ and Δ) over multiple measurements were performed to analyze the central tendency and variability in the data. A linear regression was then performed. This can be used to model the relationship between Ψ and Δ and other variables, such as thickness or refractive index, helping to understand how these parameters interact. A fit test was carried out to assess how well the experimental data fit the chosen optical model, thereby evaluating the validity of the model parameters derived from Ψ and Δ. The error must be less than 10%, and the number of measurements per sample varies between 6 and 10.

To improve the fit to the experimental data, addition of surface roughness, modelled as a thin surface layer consisting of 50% underlying material and 50% air, was performed by evaluating the Mean Squared Error (MSE) between the fit and experiment. Surface roughness can cause light scattering and alter the measured ellipsometry angles (Ψ and Δ), leading to an inaccurate determination of the refractive index. A common approach is to model the surface roughness as a layer with optical properties different from the bulk material. This layer is often described using a “graded” or “effective medium” approximation, where the refractive index gradually transitions from the air to the bulk value of the material. This helps to correct the measured values. One way to correct for these surface layers is to incorporate them into the ellipsometry model as additional thin layers above the enamel material. By including the thickness and refractive index of this layer, the correct refractive index of the enamel can be determined. In this case, however, the heterogeneity of enamels does not allow for the layer to be identified. If the enamel is applied to a substrate, the optical properties of the substrate may affect the ellipsometric measurement. The refractive index of the substrate must be included in the ellipsometry model by considering the substrate as a background layer and fitting the data to separate the contributions of the enamel and the substrate. For complex systems, where there are multiple layers (e.g., enamel, oxide layer, substrate), optical interference between the layers can complicate the ellipsometry measurement. Therefore, a Bruggeman effective medium approximation was used to account for surface roughness in the data fitting process.

**Figure 2:**
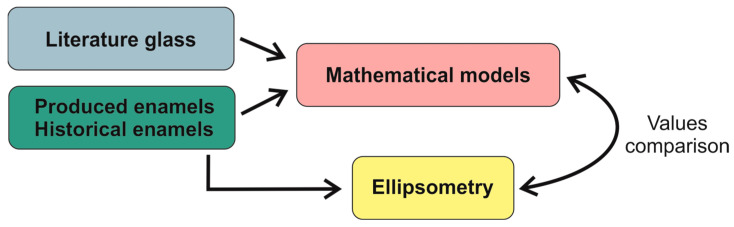
Schematic representation of the methodology used in this study.

The experimental phases considered in this study are as follows: theoretical calculations from literature models, determination of refractive index via ellipsometry, and comparison of data. The comparison between the analyzed results and the mathematical methods was made by directly comparing the theoretical value with its real value. The methodology followed in this study is shown in Figure 2.

## 3. Results and Discussion

### 3.1. Theoretical Calculations

**Figure 3:**
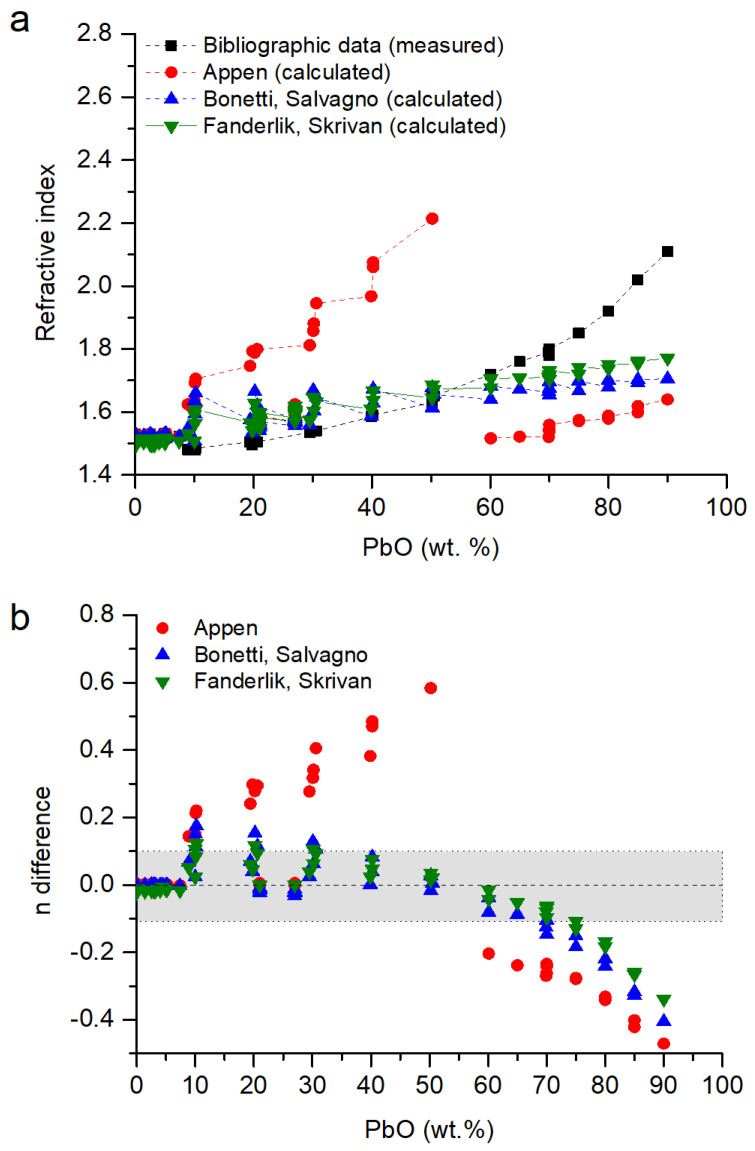
(**a**) Refractive index of lead borosilicate glasses. Data from [34,35,38,41] compared with data calculated following the Appen model [1], the Bonetti and Salvagno model [35] and the Fanderlik and Skrivan model [34]. (**b**) Difference in the values of the refractive index following n_difference_ = n_model_ − n_measured_. In grey, the values with n ± 0.1.

In Figure 3a, the refractive index of the glasses is represented as a function of the concentration of the lead oxide, as it is the most polarizable ion in the glass matrix. It is observed that the concentration of lead oxide in the glass is directly related to its refractive index. A higher concentration of PbO means a higher refractive index, especially for [PbO] > 70 wt.%.

From the three models (Section 2.3), the Appen model overestimated the refractive index by up to 0.6 units for concentrations with 20–50 wt.% PbO, and for concentrations > 60 wt.%, it was underestimated by up to 0.4 units (Figure 3b). The models proposed by Fanderlik and Skrivan and Bonetti and Salvagno are similar; they have a very good agreement for [PbO] < 10 wt.%, with errors between them of ±0.1 for glasses with chemical compositions with [PbO] < 70 wt.%. For glasses with [PbO] > 70 wt.%, the refractive index was progressively underestimated (Figure 3b). There was no good model for [PbO] > 60 wt.% because the proposed ones are focused on glasses with [PbO] < 30 wt.%, and the high polarizability of lead ions significantly increases the refractive index [2].

### 3.2. Experimental Results

**Figure 4:**
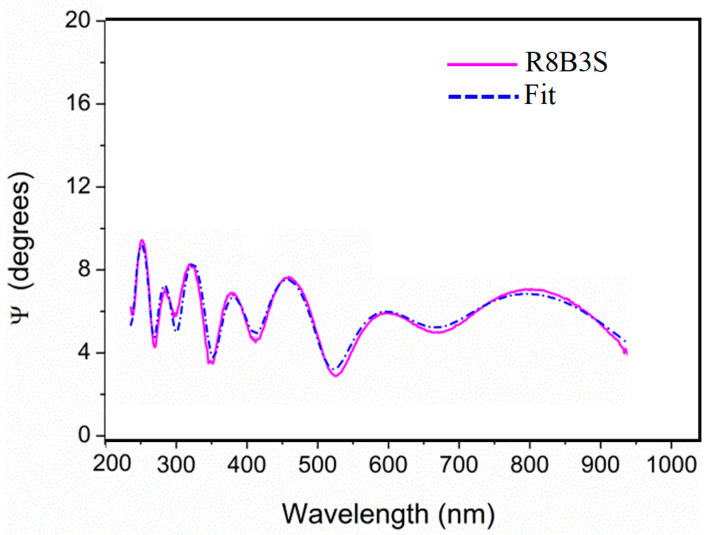
Adjustment in the ellipsometry measurement of the sample R8B3S. It is representative of all the samples analyzed.

The enamels prepared in the Ateliers, sets 1 and 2, were measured without problems using spectral ellipsometry, and the measurements were fitted using the Cauchy model [39]. Figure 4 shows the adjustment in the fitting of the measurement of sample R8B3S, which is representative of all the samples analyzed. The historical stained glasses were more difficult to measure. Some of the historical samples presented a very thin layer of grisaille, which is formed by metallic particles embedded in a lead glass matrix that can scatter the light, compromising the analysis [42]. Additionally, in general, they show an altered surface. The irregularities in the surface can also affect the measurement [43].

The refractive index of the different enamels was measured at between 1.5 and 1.7 (Table 1). Due to the large variability in the enamel matrixes, their refractive index was not as linear as the Geller and Bunting data (Figure 3a).

**Figure 5:**
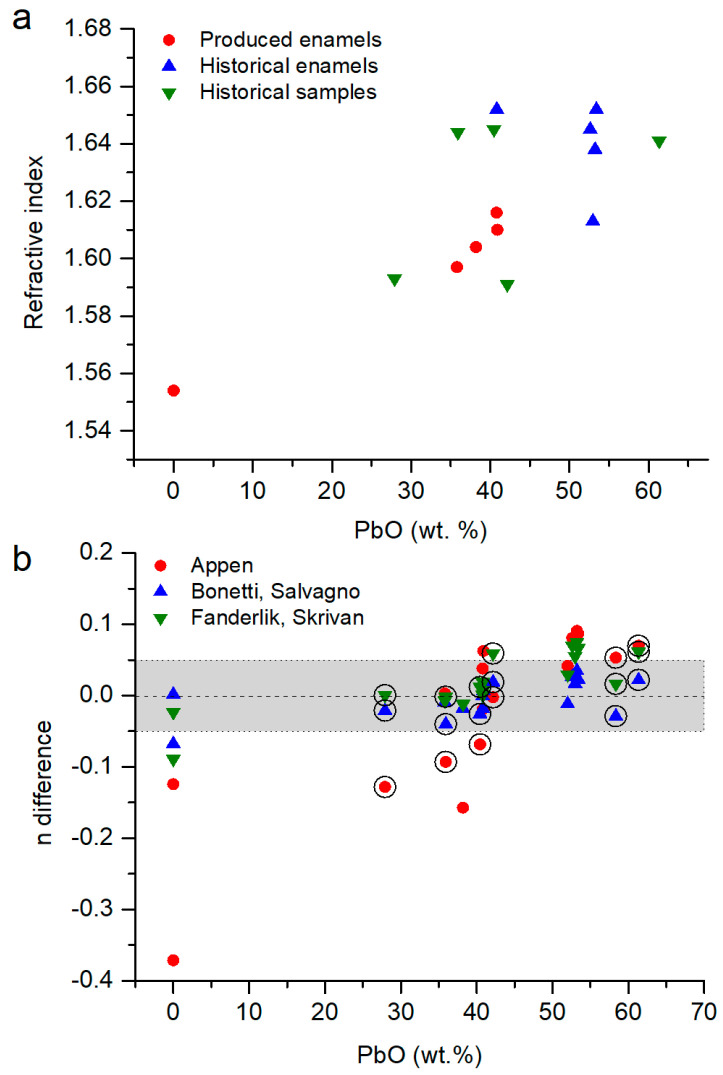
(**a**) Representation of the refractive index in function of the PbO content on the samples from sets 1, 2 and 3. (**b**) Difference between the refractive index value (n_difference_ = n_model_ − n_measured_) calculated following the Appen model [1], the Bonetti and Salvagno model [35] and the Fanderlik and Skrivan model [34] model and measured via ellipsometry (Table 1). The circles indicate the altered historical enamels. In grey, the values with n ± 0.05.

In the produced enamels (set 1), the refractive index was between 1.489 and 1.616 (Table 1, Figure 5a). Samples R6B2Z and R6B2S were prepared with six parts of base glass and one part of coloring agent [31]. These proportions of raw materials produced the highest content of PbO and, therefore, the highest refractive index. Samples R8B1S, R8B2S, and R8B3S were made with four parts of base glass and one part of coloring agent, but each sample was made with different base glasses. Sample R8B1S was made with a lead glass without borax, sample R8B2S with a lead glass with borax, and sample R8B3S with common flint glass and borax [31]. The addition of borax diminished the refractive index of the enamels, decreasing up to 0.07 units (Table 1). Finally, sample R10B3S was prepared with five parts of base glass, one part of coloring agent, and one part of copper [31]. It had a relatively high refractive index value because the enamel contains 16.3 wt.% of CuO, which compensates for the content of lead.

The three mathematical models showed average variations of 0.4 units. The model of Bonetti and Salvagno showed the most similar result to the measured data (Figure 5b). Samples R8B3S and R10B3S have the highest error because they did not have lead oxide in their composition (Table 1). In addition, none of the models include CuO in their calculations (Equations (1)–(3)), increasing the error in sample R10B3S.

The historical enamels, from set 2, show a good correlation between the PbO content and the measured refractive index (Figure 5a). Samples E3, E14, E107, E119, and E131 are chemically similar; however, their refractive indices are slightly different (Table 1). Sample E23 had less PbO, but it was compensated by a lower content of B_2_O_3_. In the case of sample E3, the high content of SiO_2_ decreased the refractive index of the enamel, even its high content of PbO. Samples EN1A and EN1C showed the highest errors in the average results of ellipsometry (Table 1), because a thick layer of grisaille was applied over the enamel, and the PbO content was low [37]. Regarding the accuracies of the models, they were overestimated by up to 0.1 unit (Figure 5b), with the largest errors in the Appen model. The model of Bonetti and Salvagno was the most accurate, with an error < 0.04 units.

Finally, six historical enamels from stained-glass windows were measured (set 3). They have chemical compositions similar to some of the enamels produced nowadays (sets 1 and 2); however, they have slightly higher refractive indices (Table 1, Figure 5a). This behavior could be due to two reasons: the presence of a thin layer of grisaille on the enamel or the formation of an alteration layer.

The presence of metallic particles from the grisaille over the enamel layer can alter the measured data. The refractive index of the most common grisaille’s particles is Fe_2_O_3_: 2.91, Fe_3_O_4_: 2.42, MnO_2_: 2.13, SiO_2_: 1.544 [44,45]. Most of these particles have refractive indices higher than simple enamels (Table 1, sets 1 and 2), so they could increase their value.

Another reason could be the alteration in the surface due to its interaction with the environment. Analysis of the alteration products in the surface revealed compounds enriched in lead [37] that could be lixiviated from the glassy matrix. This loss would decrease the refractive index of the enamel due to a reduction in the electronic density in the glass, which contradicts the experimental results.

Previous studies have investigated the change in the refractive index due to the formation of an alteration layer. Lind and Hartman [46] assessed different soda-lime silicate glasses exposed to a semi-arid environment for more than 30 years. In these glasses, the refractive index was diminished due to the formation of alteration layers of 148–228 nm. Similarly, Casparis-Hauser and Guenther [47] also observed a decrease in the refractive index due to the formation of an alteration layer on the surface of a glass enriched in BaO and ZnO (N-BAK4). Kaspar et al. [40] characterized the effect of induced corrosion on a borosilicate glass (ISG glass), and they also observed that the refractive index of the alteration layer was lower than the glass substrate. Only in a study by Portal and Sempere [48] was the formation of a two-layer system observed in a soda silicate glass with a higher refractive index, which was attributed to the densification of the leached layer.

Therefore, the increase in the refractive index of the enamels could be due to the application of the thin layer of grisaille on the surface of the enamels.

For these samples, the mathematical models from Fanderlik and Skrivan and Bonetti and Salvagno agreed with the experimental results (Figure 5b). The error in the Fanderlik and Skrivan model was up to 0.06 units, but the error in the model of Bonetti and Salvagno was <0.04 units. The latter model was developed for “sonoro superiore” lead glasses with 21–27 wt.% PbO. Nevertheless, it also works for glasses with a higher content of lead.

Very few studies have analyzed the refractive index of historical glasses, and none of them characterized the glazed surface paintings. The historical lead glasses analyzed by Tennent and Townsend [10] had refractive indices between 1.550 and 1.570; however, the chemical composition of these pieces has not been published. Vassas [11] did not analyze lead glasses, and Saminpanya et al. [12] did not publish the value of each glass bead, so it is not possible to compare the n value with the chemical composition. Finally, Drozdov et al. [13] analyzed some opaque potash-lead silicate glasses with n values between 1.493 and 1.825. For a similar lead content (sample 1), the n was 1.825, a higher value than that obtained in the present study, but its chemical composition did not present boron, unlike the historical enamels. This light element reduces the value of the refractive index [2].

## 4. Conclusions

In general, enamels and grisaille paints have a higher refractive index (1.59–1.66) than historical glasses due to their high lead oxide content. When compared with historical lead glass, they have higher values than measured in the literature (1.55–1.57), probably because the surface paints contain metallic elements that increase the electronic density of the glass and, therefore, the n value.

This study evaluated the accuracy of three mathematical models. The Appen model was found to be less accurate than the models proposed by Bonetti and Salvagno and by Fanderlik and Skrivan. The latter is similar, with the Bonetti and Salvagno model being slightly better because it was developed for glasses with a higher PbO content. Nevertheless, the three models do not work for glasses with >60% PbO, which are not common in cultural heritage.

This study also showed that ellipsometry is a suitable technique for analyzing the refractive index of glass and surface paints without damaging the stained-glass window’s fragments. A limitation of this technique is that historical samples are not homogeneous, so a large number of measurements are needed to reduce the error. In addition, the presence of very thin surface layers such as grisaille or alteration layers inherent to the cultural object can also modify the refractive index value, requiring more measurements to obtain an average value. Polishing or damaging the object is not allowed under any circumstances.

Future lines of research include the application of ellipsometric analyses to samples of different chronologies, locations, and chemical compositions to provide a database useful for restorers and heritage scientists.

## Figures and Tables

**Table 1:** Refractive index and thickness of surface enamels measured via ellipsometry.

Set	Sample	n Calculated			Results Ellipsometry		Main Elements of the Chemical Composition (wt.%)									
		Appen	Bonetti, Salvagno	Fanderlik, Skrivan	Refractive Index	B_2_O_3_	Na_2_O	MgO	Al_2_O_3_	SiO_2_	K_2_O	CaO	CuO	ZnO	BaO	PbO
Set 1. Produced enamels [31]	R6B2Z	1.65	1.62	1.62	1.616 ± 0.004	10.9	4.7	-	-	22.4	15.8	-	-	-	-	40.8
	R6B2S	1.67	1.62	1.62	1.610 ± 0.011	11	4.7	-	-	22.6	16.3	-	-	-	-	40.9
	R8B1S	1.45	1.59	1.59	1.604 ± 0.006	-	3.7	-	0.2	34.3	17.9	-	-	-	-	38.2
	R8B2S	1.6	1.59	1.59	1.597 ± 0.011	5.1	2.2	-	0.2	34.1	17.2	-	-	-	-	35.8
	R8B3S	1.36	1.49	1.47	1.489 ± 0.009	1.3	4.3	-	0.2	71.2	17.3	-	-	-	-	-
	R10B3S	1.18	1.49	1.46	1.554 ± 0.063	1.1	3.8	-	0.1	59.4	14.6	-	16.3	-	-	-
Set 2. Replica enamels [36]	E3	1.68	1.63	1.67	1.613 ± 0.003	7	5.1	0.1	0.3	30.4	0.1	2.6	-	-	-	53
	E14	1.73	1.67	1.71	1.638 ± 0.004	21.1	2.1	0.1	0.3	8.6	0.2	0.9	-	11.2	-	53.2
	E23	1.63	1.63	1.67	1.652 ± 0.007	16.6	0.8	-	0.3	9.3	0.1	0.5	-	14.0	-	40.8
	E107	1.73	1.67	1.72	1.645 ± 0.003	19.8	0.9	-	0.2	8.9	0.3	0.6	-	13.8	-	52.6
	E119	1.74	1.67	1.72	1.652 ± 0.003	20.4	1.1	-	0.2	8.2	0.3	0.5	-	13.9	-	53.4
	E131	1.73	1.67	1.71	1.684 ± 0.002	21.3	1.5	0.1	0.5	9.3	0.1	0.7	-	12.9	-	52.0
Set 3. Historical 20th cent. stained-glass windows [37]	BC3B blue	1.59	1.61	1.65	1.591 ± 0.008	5.2	4.9	0.1	3.6	28.3	0.1	1.2	-	8.1	0.2	42.2
	BC3C green	1.58	1.62	1.66	1.645 ± 0.003	7.5	6.3	0.1	1.9	26.5	0.2	7.1	1.6	2.2	0.1	40.5
	PG2C bluish	1.55	1.6	1.64	1.644 ± 0.006	4.5	5.5	0.1	2.1	32.9	0.1	7.2	1.9	3.7	0.4	35.9
	PG2C purple	1.46	1.57	1.59	1.593 ± 0.005	3.5	6.7	0.2	0.7	41.6	0.3	5.8	-	0.2	0.4	27.9
	EN1A green	1.71	1.66	1.7	1.641 ± 0.015	15.7	1.4	-	0.8	15.8	0.1	0.8	1.6	1.9	-	61.3
	EN1C yellow	1.75	1.66	1.71	1.693 ± 0.029	14.5	1.5	-	0.5	15	0.1	1.0	-	7.5	-	58.4

## Data Availability

The data that support the findings of this study are available from the corresponding author upon reasonable request.

## Abbreviations

k	extinction coefficient
MPEF	Multiphoton excitation fluorescence microscopy
MSE	Mean Squared Error
n	Refractive index
NLOM	Nonlinear optical microscopy
OCT	Optical coherence tomography
THG	Third-harmonic generation microscopy
THz-TDS	Terahertz time-domain spectroscopy
wt.%	Weight percent
